# Isolation and Characterization of a New Lytic Phage MA9V-2 Against *Chryseobacterium indologenes* MA9 and Its Combined Application with MA9V-1 for the Control of *Panax notoginseng* Root Rot

**DOI:** 10.3390/microorganisms14071423

**Published:** 2026-06-29

**Authors:** He Zou, Juncen Liu, Yizhi Ye, Jun Liu

**Affiliations:** 1 School of Food and Liquor Engineering, Sichuan University of Science & Engineering, Zigong 643000, China; 18381307626@163.com (H.Z.); 18981753225@163.com (J.L.); 17606684980@163.com (Y.Y.); 2 Key Laboratory of Brewing Biotechnology and Application of Sichuan Province, Zigong 643000, China

**Keywords:** *Chryseobacterium indologenes* MA9, *Panax notoginseng* root rot, myoviridae, phage biocontrol, whole-genome analysis

## Abstract

*Panax notoginseng*, a valuable medicinal plant in Yunnan, suffers significant yield losses due to root rot, with *Chryseobacterium indologenes* MA9 as a major causal agent. Conventional chemical control methods are limited by residues and the development of bacterial resistance, underscoring the need for alternative strategies. In this study, we isolated a new lytic myovirus, vB_CinP_MA9V-2, from wastewater using MA9 as the host. MA9V-2 exhibited high lytic efficiency with 75% adsorption in 25 min and a burst size of ~100 PFU per cell, stability across pH 4 to 11 and temperatures of 4 to 50 °C, a moderately broad host range, and effective suppression of biofilm formation. Genome analysis confirmed the absence of virulence or antibiotic resistance genes, indicating its safety for application. In potted plant experiments, single-phage treatment reduced root rot incidence to 16.7% compared with 83.3% in the control, while a combined treatment with phages achieved up to 80 percent control. Curative effects post-infection were limited with a disease incidence of 61.3%, highlighting the preventive advantage of phage therapy. Overall, these results demonstrate that phage therapy, particularly using a combination of phages, shows potential for application in the management of bacterial root rot in *P. notoginseng*.

## 1. Introduction

*Panax notoginseng* is a rare and highly valued traditional Chinese medicinal plant, with its primary production area located in Wenshan Prefecture, Yunnan Province, China. The annual output value of the *P. notoginseng* industry exceeds 10 billion yuan, reflecting its considerable economic and medicinal significance [1]. The roots of *P. notoginseng* are rich in bioactive compounds, including saponins and polysaccharides, which serve as key raw materials for over 400 pharmaceutical products, such as Xuesaitong capsules and Yunnan Baiyao [2]. However, continuous cultivation of *P. notoginseng* has caused severe disturbances to the soil micro-ecosystem, resulting in “continuous cropping obstacles”. Among these, root rot is the most destructive disease. This complex disease is caused by the combined activity of multiple soil-borne pathogens, including fungi such as *Fusarium oxysporum* [3], bacteria [4], and nematodes. This disease can cause the leaves of the growing-stage *Panax notoginseng* plants to wither and the roots to rot, resulting in annual yield losses of 5–20%, while severe outbreaks can cause losses exceeding 70% or even complete crop failure, thereby constituting a major bottleneck restricting the sustainable development of the *P. notoginseng* industry. Previous studies on root rot of *P. notoginseng* have primarily focused on fungal pathogens, particularly *Fusarium oxysporum* [3,5,6]. In recent years, however, accumulating evidence has highlighted the significant role of bacterial pathogens in disease development. Notably, *Chryseobacterium indologenes* MA9 has been identified as an independent bacterial pathogen responsible for root rot in *P. notoginseng*, challenging the long-standing assumption that bacteria act solely as secondary or synergistic agents [7,8,9]. Studies have shown that disease incidence and severity are substantially increased when *Pseudomonas*, *Fusarium*, and nematodes coexist, with bacterial involvement markedly exacerbating disease progression [10]. Although at least six bacterial pathogens associated with *P. notoginseng* root rot have been reported, investigations into the pathogenic mechanisms and effective control strategies specifically targeting MA9 remain limited [9].

Current management of *P. notoginseng* root rot relies predominantly on chemical fungicides and antibiotics. However, these approaches present several notable limitations. First, the accumulation of pesticide residues in the soil disrupts the native microbial community, leading to environmental contamination and long-term ecological imbalance [11]. Second, the emergence of antimicrobial resistance poses a significant challenge; for instance, *Chryseobacterium indologenes* MA9 exhibits resistance rates of 95–100% to cephalosporins and carbapenems, with only limited susceptibility to compound sulfamethoxazole (resistance rate of 16.7%) [12]. Third, conventional chemical treatments typically target individual pathogens and are insufficient to address the multifactorial nature of root rot, which involves complex interactions among fungi, bacteria, and nematodes. These limitations highlight the urgent need for novel, effective, and environmentally sustainable strategies for controlling *P. notoginseng* root rot.

Phages have emerged as environmentally friendly, cost-effective, and sustainable agents for managing bacterial plant diseases [13]. For example, the *Ralstonia solanacearum* phage RsoM1USA, isolated from tomato fields in Florida, represents a novel species within the family Peduoviridae and effectively delays wilting in tomato plants at a multiplicity of infection of 0.01 [14]. Similarly, the myovirus PHB09 exhibits potent lytic activity against *Pseudomonas syringae* pv. actinidiae both in vitro and in vivo, demonstrating its potential as a biocontrol agent to mitigate crop losses [15]. Moreover, phage-based combination therapies can effectively suppress bacterial wilt in tomato, and soil microbiome analyses conducted before and after phage treatment revealed that phages may enhance plant health by modulating the relative abundance of pathogens and phage-resistant strains, providing mechanistic insight into phage-mediated suppression of bacterial diseases [16].

The widespread misuse of antibiotics has accelerated the emergence and dissemination of resistant pathogens, highlighting the urgent need for alternative antibacterial strategies. Phage therapy has emerged as a particularly promising approach due to several advantages. First, phages exhibit high diversity, inhabiting nearly all ecosystems and constituting a vast natural reservoir for therapeutic development [17]. Second, phages display high specificity and safety, as most infect only a single bacterial species without adversely affecting non-target microbes or host cells [18,19]. Third, phages offer high efficacy, with rapid replication and large burst sizes enabling effective pathogen suppression at relatively low dosages. Fourth, phages demonstrate adaptability, as long-term coevolution with their bacterial hosts drives reciprocal modifications in bacterial receptors and phage adsorption or DNA-modification mechanisms [20,21]. Although phage therapy has been explored for the control of several plant bacterial diseases, its application against *Chryseobacterium indologenes*-induced root rot in *P. notoginseng* has not been reported. Moreover, the interactions between phages and this pathogen remain poorly understood.

*C. indologenes* MA9 is a bacterial pathogen capable of independently causing severe root rot disease in *P. notoginseng*. Although conventional control measures, such as pesticides and antibiotics, can effectively suppress the disease, their extensive use often results in chemical residues and the emergence of antibiotic-resistant bacterial populations, thereby posing increasing challenges for sustainable disease management. Consequently, phages, owing to their high specificity, strong bactericidal efficacy, and capacity for self-amplification, have emerged as a promising alternative for the biological control of bacterial plant diseases. In the present study, we investigated root rot of *P. notoginseng* caused by *Chryseobacterium indologenes* MA9 and, for the first time, explored a phage-based biocontrol strategy targeting this pathogen. Pathogenicity assays were conducted to confirm the virulence of MA9 in two-year-old *P. notoginseng* plants. Subsequently, lytic bacteriophages specific to MA9 were isolated from sewage samples and characterized biologically to evaluate their infectivity, lytic activity, and host specificity. To assess their disease control potential, both individual phage treatments and phage cocktail formulations were evaluated in greenhouse pot experiments. Particular emphasis was placed on determining whether phage cocktail therapy could achieve synergistic effects, resulting in greater disease suppression than that obtained with individual phages alone.

Furthermore, whole-genome analyses of the isolated phages were performed to identify genes potentially involved in phage-host interactions, including receptor-binding proteins and lysis-related systems. These analyses provide preliminary insights into the molecular mechanisms underlying phage-mediated suppression of *P. notoginseng* root rot. In this study, we evaluated the potential of phage-based treatments, including phage cocktail formulations, for the biocontrol of *P. notoginseng* root rot caused by *Chryseobacterium indologenes* MA9. We further investigated whether combined phage application could improve disease suppression compared with single-phage treatments.

## 2. Materials and Methods

### 2.1. Experimental Materials

The host strain *Chryseobacterium indologenes* MA9 (GenBank accession no. CP075170) was provided by Professor Ji’s team at the College of Plant Protection, Yunnan Agricultural University. Phage MA9V-2 was isolated from a sewage sample collected in the sewer at the First People’s Hospital of Yunnan Province. Phage MA9V-1, screened with *C. indologenes* MA9 as host strain, is a myovirus phage with strong lytic ability previously obtained in the early stage of this study [22]. The sampled *P. notoginseng* plants were healthy two-year-old specimens. The sampling site was located in Shilin Yi Autonomous County, Kunming, Yunnan Province (103°15′40.576″ E, 24°44′25.004″ N).

### 2.2. Phage Isolation, Purification, and Enrichment

Initial screening for phages targeting *C. indologenes* MA9 was conducted using viral enrichment and the double-layer agar method from four different sample sources [23]. Ultimately, a phage named MA9V-2 was successfully isolated from the hospital sewage sample [24]. The protocol was as follows: sewage samples were first filtered using a 0.22 μm membrane filter (Beijing Lanjieke Technology Co., Ltd., Beijing, China). Then, 50 mL of the filtrate was mixed with 50 mL of SM buffer (100 mM NaCl, 10 mM MgSO_4_, 50 mM Tris-HCl at pH 7.5, and 0.01% gelatin, 1 L) in a 250 mL Erlenmeyer flask and left undisturbed for 12 h. Subsequently, 40 mL of the clarified liquid was combined with 30 mL of SM buffer, 25 mL of Nutrient Broth medium with a modification (NB, peptone 5 g, beef extract 3 g, yeast extract powder 1 g, 1 L), and 2 mL of *C. indologenes* MA9 culture, followed by overnight incubation on a horizontal rotating oscillator (Kylin-Bell Lab Instruments Co., Ltd., Nantong, China) at 28 °C and 160 rpm. The mixture was then centrifuged at 15,000× *g* for 15 min at 4 °C (Xiangyi Centrifuge Instrument Co., Ltd., Changsha, China), and the supernatant was filtered again using a 0.22 μm membrane. For enrichment, 5 mL of the filtrate was added to 50 mL of log-phase *C. indologenes* MA9 culture (OD_600_ = 0.6–0.8) and incubated for 24 h. This enrichment process was repeated 2–3 times. Subsequently, 200 μL of phage suspension was serially diluted with SM buffer, and each dilution was mixed with 300 μL of *C. indologenes* MA9, incubated at 28 °C for 20 min, and added to 4.5 mL of semi-solid NB medium. The mixture was poured over solid NB plates for plaque formation.

The methods for enriching and purifying phages are as follows [22]: From NB double-layer agar plates incubated overnight at 28 °C, single clear plaques were picked and placed into 1 mL SM buffer, vortexed for 30 s, and filtered through a 0.22 μm membrane. This purification step was repeated 2–3 times to obtain a pure MA9V-2 phage stock. The phage titer was determined to be 10^10^ PFU/mL using the double-layer agar method and stored at 4 °C.

### 2.3. Optimal Multiplicity of Infection

The optimal multiplicity of infection (MOI) was defined as the ratio of phage particles to host bacterial cells at the time of infection [25]. Log-phase *C. indologenes* MA9 cultures were serially diluted to 10^−4^, 10^−5^, and 10^−6^, and the CFU/mL was calculated using the dilution plating method. Different MOI values (0.001, 0.01, 0.1, 1, and 10) were tested by mixing known titers of MA9V-2 phage with MA9 host cells in log phase and incubating the mixtures at 28 °C on a shaker at 180 rpm for 12 h. After incubation, the cultures were centrifuged at 15,000× *g* for 15 min at 4 °C and filtered through 0.22 μm membranes. Phage titers were determined using the double-layer agar method. Each experiment was conducted in triplicate. A one-way analysis of variance (ANOVA) was applied for statistical analysis in this study.

### 2.4. Adsorption Rate Assay

To determine the adsorption kinetics of MA9V-2 to its host [26], 1 mL of diluted phage suspension (10^6^ PFU/mL) was mixed with 9 mL of log-phase *C. indologenes* MA9 (OD_600_ = 0.6–0.8) at an MOI of 0.01. The mixture was incubated at 28 °C. Samples (approximately 100 μL) were taken every 2 min, immediately added to 1.9 mL of pre-chilled NB medium in sterile tubes, vortexed for 10–15 s, and centrifuged at 15,000× *g* for 15 min at 4 °C to remove adsorbed phages. The number of unadsorbed (free) phages in the supernatant was quantified using the double-layer agar method. Each experiment was conducted in triplicate.

### 2.5. One-Step Growth Curve

To determine the latent period and burst size of MA9V-2, one-step growth assays were performed [27]. Log-phase *C. indologenes* MA9 cultures (10^8^ CFU/mL) were centrifuged at 11,000× *g* for 8 min at 4 °C, and the pellet was resuspended in 2 mL of fresh NB medium. MA9V-2 phage was added at MOI = 0.01, in which the host bacteria were determined using the plate count method, and the phage titer was measured using the double-layer agar plate method, and the mixture was incubated at 28 °C for 15 min to allow adsorption. The mixture was then centrifuged to remove unadsorbed phages and washed twice with fresh NB medium. The pellet was resuspended in 10 mL NB medium and incubated at 28 °C with shaking at 180 rpm. Samples were taken every 10 min, centrifuged at 15,000× *g* for 3 min, and titers were determined using the double-layer agar method. Burst size was calculated as the ratio of total phage particles released to the initial number of infected host cells. Each experiment was conducted in triplicate.

### 2.6. Thermal and pH Stability Assays

To assess thermal stability [25], 1 mL of MA9V-2 lysate (10^10^ PFU/mL) was incubated at various temperatures (4 °C, 28 °C, 40 °C, 50 °C, 60 °C, and 70 °C) for 1 h. Phage viability was determined by plaque assay. For pH stability, 100 μL of phage suspension was added to 900 μL of buffer solutions with pH values ranging from 3 to 12 (prepared with sodium citrate, potassium dihydrogen phosphate, Tris-HCl, and sodium carbonate). After 1 h of incubation at 30 °C, phage titers were determined as above. Each experiment was conducted in triplicate.

### 2.7. Host Range Assays

The host range of MA9V-2 was assessed using the spot assay against 11 bacterial strains, including *C. indologenes* (*n* = 7, 6 strains were selected from soil, sewage and mud), *Bacillus cereus* (*n* = 1), *Pseudomonas syringae* (*n* = 1), and *Escherichia coli* (*n* = 1). Three strains (*C. indologenes* ATCC 29897, *P. syringae* CGMCC 1.3070, and *E. coli* ATCC 11303) were purchased from the strain preservation center, and the others were preserved by our laboratory. For each test, 300 μL of bacterial culture was mixed with 200 μL of serially diluted MA9V-2 (10^−1^ to 10^−7^), then added to 4.5 mL of semi-solid medium and poured onto NB agar plates. After overnight incubation at 28 °C, the presence of plaques was observed. In addition, a double-layer culture plate without adding phages was used as the negative control, while the susceptible host strain *C. indologenes* MA9 was used as the positive control. Each experiment was conducted in triplicate. The calculation method of efficiency of plating is as follows:

EOP (Efficiency of Plating) = (PFU on test host)/(PFU on reference host).

### 2.8. Phage Transmission Electron Microscopy

Phages were purified by CsCl density gradient centrifugation as follows [22]: (1) Using SM buffer as the solvent, three CsCl solutions with different densities (1.45, 1.50, and 1.70 g/mL) were prepared. A total of 1.5 mL of each CsCl solution was sequentially layered from highest to lowest density into an ultracentrifuge tube. (2) The volume of the extracted aqueous phase was measured, and CsCl was added at a ratio of 0.5 g/mL. The mixture was gently stirred with a pipette tip until the CsCl was completely dissolved, and 4.5 mL of the solution was carefully layered onto the top of the gradient. (3) After centrifugation at 4 °C and 150,000× *g* for 10 h, a distinct white opalescent band was observed. Approximately 0.5–1 mL of the phage-containing band was collected using a 1 mL syringe inserted through the side of the centrifuge tube and transferred to a 2 mL microcentrifuge tube, sealed with parafilm, and stored at 4 °C. Phage particles purified by density gradient centrifugation were adsorbed onto carbon-coated copper grids by spotting 10 μL of sample. After 10 min of adsorption, grids were negatively stained with 1% phosphotungstic acid. The morphology of MA9V-2 was observed using a transmission electron microscope (Hitachi HT7820, Tokyo, Japan) at 120 kV [28].

### 2.9. Genome Sequencing, Annotation, and Bioinformatic Analysis

To improve genome purity, 2.5 μL of DNase I (1 U/μL) and 0.5 μL of RNase A (1 mg/mL) were added to 500 μL of phage stock to eliminate host nucleic acids. The purified DNA was extracted using a phenol-chloroform protocol. DNA concentration was measured using a NanoDrop spectrophotometer (BIO-DL, Shanghai, China), and quality was assessed by 1% agarose gel electrophoresis [29].

Whole-genome sequencing of the phage was performed using the Illumina NovaSeq PE150 platform (Huada Intelligent Manufacturing Technology Co., Shenzhen, China). Genome assembly and analysis were conducted using multiple bioinformatics tools. Proksee was used to construct the circular genomic maps. HHpred was applied to predict open reading frames (ORFs) and annotate their biological functions. Comparative analyses were conducted with NCBI databases using ClustalW v2.1 [30]. Phylogenetic trees were generated using ViPTree v1.9 (for proteomic trees) and MEGA v11 (for specific ORFs) [31,32]. Easyfig v2.2 was used for multiple genome alignment and homology visualization, and VIRIDIC(v1.1) was applied to calculate genomic similarity between MA9V-1 and MA9V-2 [33,34]. Finally, the presence of virulence factors and antibiotic resistance genes was screened using relevant online databases to evaluate biosafety for phage application [35].

### 2.10. Pathogenicity Analysis, Disease Scoring and Pathogenicity Assessment of C. indologenes MA9

Healthy two-year-old *P notoginseng* seedlings at the vegetative growth stage, approximately 35–50 cm in height, were transplanted into pots (17 cm × 15 cm, 3.4 L). The seedlings were then covered with a commercial potting substrate amended with perlite and zirconite (Lingshou Ningbo Mineral Products Co., Ltd., Shijiazhuang, Hebei, China), with the substrate filling approximately two-thirds of the pot volume. Placing the potted plants in a room temperature environment (25 °C), with a relative humidity (RH) of 40–60%, and were grown under a 16 h light/8 h dark photoperiod (Light intensity ranges from 160 μmol·m^−2^·s^−1^ to 300 μmol·m^−2^·s^−1^.). After 7 days of growth, the conditions should remain stable. The negative control group was treated with NB medium, and the positive group was inoculated with *C. indologenes* MA9 in its logarithmic phase. Two different inoculation methods, root drenching (GG) and foliar spraying (PW), were employed with three volumes of bacterial suspension (10 mL, 20 mL, and 50 mL) (5.0 × 10^8^ CFU/mL). Spraying was selected to simulate practical field conditions, as it allows phages to come into contact with aerial parts that may indirectly influence root infection and mirrors methods commonly used in agricultural applications. Root drenching was included to directly target soil-borne pathogens within the rhizosphere and ensure uniform exposure of the roots to phages, which are the primary infection sites for root rot. By comparing these two application methods, we were able to assess both the preventive and curative potential of phage therapy and determine the most effective delivery strategy. Two additional bacterial strains, *Pseudomonas syringae* PSS (a pathogenic bacterium) and *Bacillus subtilis* BYM 41-22 (a non-pathogenic strain), were used as controls. The plants were inoculated with the bacterial suspensions every 24 h, and after 7 days, the plants were examined for disease symptoms. Each treatment group consisted of 3 independent pots, with one *P. notoginseng* plant per pot. All measurements were performed on each individual plant. Therefore, the experiment included 3 biological replicates, with each measurement conducted in technical triplicate. This design was consistently applied in all subsequent pot experiments to ensure the reliability and reproducibility of the results. All treatment groups included three replicate plants, which were randomly selected, and the positions of the pots were randomly rearranged every 24 h to minimize environmental variation.

The degree of leaf wilting was used to categorize the severity of disease in plants into five levels, with scores ranging from 0 to 4: 0 = no symptoms; 1 = mild wilting (1% to 25% of leaves affected, with possible recovery); 2 = moderate wilting (26% to 50% of leaves affected, with partial recovery possible); 3 = severe wilting (51% to 75% of leaves affected); 4 = extensive wilting (more than 75% of leaves affected, leading to leaf death and plant mortality). The definition of the term “partial recovery” is that after the appearance of disease symptoms, the use of the pathogenic bacteria suspension treatment is stopped, allowing the plant to have the possibility of stopping the delay of the lesion, stopping the lesion, and even restoring its own healing ability to the initial growth state.

Disease incidence (DI) was calculated as:

DI = [∑(ni × i)/(N × iMAX)] × 100%

where i represents the disease level, ni is the number of plants at disease level i, N is the total number of plants, and iMAX is the maximum disease level.

### 2.11. Phage Prevention and Biocontrol of P. notoginseng Root Rot in Pot

The pot experiment for phage prevention of root rot included five treatment groups: (1) A blank control group with 20 mL of NB medium; (2) A positive control group where plants were treated with 20 mL *C. indologenes* MA9 in its logarithmic phase; (3) MA9V-1 (1.25 × 10^10^ PFU/mL) at MOI = 0.01 mixed with 20 mL *C. indologenes* MA9, after mixing with the pathogen, the phage was incubated in a 28 °C constant temperature incubator for 15 min, and then the spraying treatment was carried out, subsequent processing is the same.; (4) MA9V-2 (9.84 × 10^9^ PFU/mL) at MOI = 0.01 mixed with 20 mL *C. indologenes* MA9; (5) A combination of MA9V-1 and MA9V-2 at MOI = 0.01 mixed in a 1:1 ratio, the mixture was mixed with 20 mL *C. indologenes* MA9. In the post-infection biocontrol experiment, plants were first inoculated with 20 mL *C. indologenes* MA9 and, after 3 days, treated with either MA9V-1 (MOI = 0.01), MA9V-2 (MOI = 0.01), or the combination of both phages in a 1:1 ratio. Each experimental group was treated at 9:00 a.m, and the treatment was repeated every 24 h for 7 consecutive days. Plant growth and disease development were monitored throughout the experiment to evaluate disease outcomes.

Three diseased and necrotic leaves were randomly collected from each *P. notoginseng* plant in each treatment group. All leaf samples were ground in a sterile mortar and pestle and then added to 50 mL of NB medium. The suspensions were incubated in a shaking incubator at 28 °C and 180 rpm for 2 h. After incubation, the cultures were diluted and spread onto NB agar plates. After 12 h of incubation, colony-forming units (CFUs) were counted, and the bacterial load was calculated based on the dilution factor. In addition, Necrotic leaves were also collected from each group, ground using sterile mortars, and the homogenates were suspended in 10 mL of SM buffer. The suspension was incubated at 28 °C and 180 rpm for 2 h, then left to stand at room temperature for 2 h. The supernatant was collected, centrifuged at 15,000× *g* for 15 min at 4 °C, and filtered through 0.22 μm membranes. The filtrate was serially diluted, and the phage titers were determined using the double-layer agar method after overnight incubation at 28 °C. Plaques were counted to calculate phage concentration.

### 2.12. Statistical Analysis of Disease Incidence and Biocontrol Efficacy

All statistical analyses were performed using GraphPad Prism 9.0. Data are presented as mean ± standard deviation. Differences among multiple treatment groups, including single- and combined-phage treatments, pathogen CFU counts, phage titers, and disease incidence, were evaluated using one-way ANOVA followed by Tukey’s post hoc test for normally distributed data, with normality and homogeneity of variance verified prior to analysis. Statistical significance was set at *p* < 0.05.

Disease incidence (DI) was calculated as described in Section 2.10. Biocontrol efficacy (BCE) was determined using the following formula:

BCE = [(Dick − DIt)/DIck] × 100%

where t is the experimental group, DIck is the disease incidence in the control group, and DIt is the disease incidence in the treatment group.

## 3. Results

### 3.1. Isolation, Identification, and Morphological Observation of Phages

**Figure 1:**
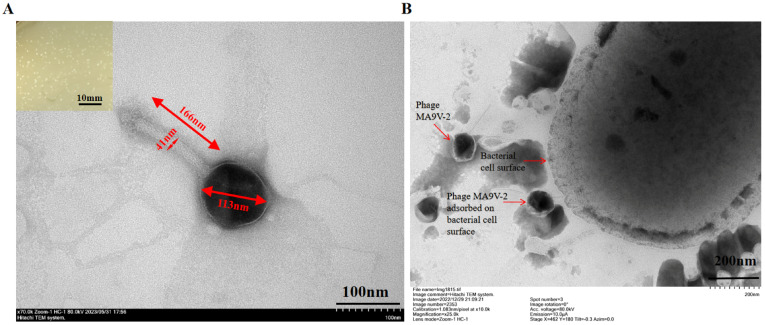
Morphological characteristics of phage MA9V-2. (**A**) Plaque size and morphology were observed under transmission electron microscopy (TEM). (**B**) Phage MA9V-2 adsorbed onto the membrane surface of its host *C. indologenes* MA9.

Using the pathogenic bacterium *C. indologenes* MA9 as the host, highly lytic phages were isolated from sewage using the double-layer agar method. According to the scientific nomenclature of the International Committee on Taxonomy of Viruses (ICTV), the new lytic phage was named vB_CinP_MA9V-2, hereafter referred to as MA9V-2. This paper focuses on the characteristics of MA9V-2 and the combined therapeutic application of both phages for the control of *P. notoginseng* root rot disease. In the determination of optimal lysis conditions, the results showed that the best lysis effect was achieved when the phage and host were incubated at a ratio of 200:300 μL, at 28 °C for 15 min, and when the mixture was mixed with semi-solid agar medium at a ratio of 1:8. Phage MA9V-2 was able to form clear, round plaques with a diameter of approximately 0.4–1.4 mm on double-layer agar plates with MA9 bacterial biofilms, and these plaques exhibited similar morphology (Figure 1A). The formation of these plaques clearly indicates the lytic capability of the phage, which is also a reflection of the phage’s ability to infect and replicate within the host.

For further characterization, the purified and concentrated phage particles were observed under a TEM. According to the latest ICTV classification system, phage MA9V-2 belongs to the Myoviridae family and is classified as a myovirus. The lytic phage MA9V-2 has an icosahedral protein “head” approximately 113 nm in diameter (Figure 1A). The length and width of the contractile tail of MA9V-2 were measured to be approximately 166 nm and 41 nm, respectively. TEM images clearly show that phage MA9V-2 attaches to the surface of the host *C. indologenes* MA9 cell membrane (Figure 1B). This indicates that the phage’s adsorption receptor is located on the host cell membrane, and the thick capsule surrounding the host cell hinders the selection of phages, which also explains the inconsistent phage selection process observed during the MA9V-1 screening.

### 3.2. Biological Characteristics of Phage MA9V-2

**Figure 2:**
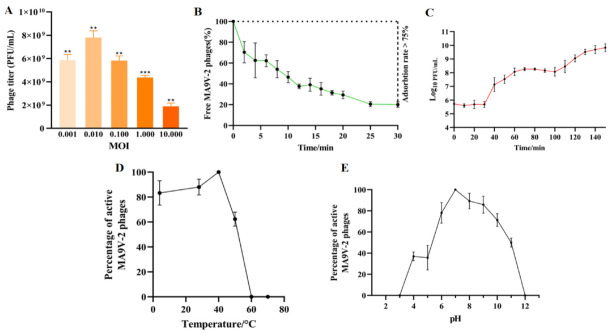
Biological features of phage MA9V-2. (**A**) The MOI of phage MA9V-2. Statistical significance is indicated as *p* < 0.01 (**), and *p* < 0.001 (***); (**B**) Adsorption curve of phage MA9V-2; (**C**) One-step growth curve of phage MA9V-2; (**D**) Relative activity of phage MA9V-2 under various temperature conditions, indicating its thermal stability; (**E**) Relative activity of phage MA9V-2 at different pH levels, reflecting its pH stability. Data are presented as mean ± SD from three independent replicates (n = 3).

The phage MA9V-2 exhibited significant variation in titer across different MOIs, with the highest titer of approximately 9.84 × 10^9^ PFU/mL observed at an MOI of 0.01, indicating this as the optimal MOI (Figure 2A). The adsorption assay showed that over 75% of MA9V-2 particles adsorbed within 25 min, demonstrating strong host affinity (Figure 2B). One-step growth curve analysis revealed a latent period of 30 min, a rise period of 40 min, and a burst size of ~100 PFU per cell, confirming MA9V-2′s lytic potential (Figure 2C). Stability tests further showed that the phage remained active between 4 and 50 °C and pH 4–11 (Figure 2D,E), aligning well with the host MA9′s optimal growth conditions (28 °C, pH 7.2). These results collectively highlight MA9V-2′s robust biological properties and its potential as a promising agent for the biocontrol of pathogenic bacteria.

### 3.3. Phage Host Range

Among 11 different host strains, MA9V-2 was found to infect *C. indologenes* MA9, *C. indologenes* 02, and *C. indologenes* 03, as well as *C. indologenes* 06, demonstrating a moderate host range (0 < EOP < 0.001), as shown in Table 1. Additionally, MA9V-2 does not infect other cross-genus bacteria, highlighting the high specificity of the phage, which ensures that it is unlikely to adversely affect non-target microorganisms in biocontrol applications.

### 3.4. Genome Characteristics of MA9V-2

**Figure 3:**
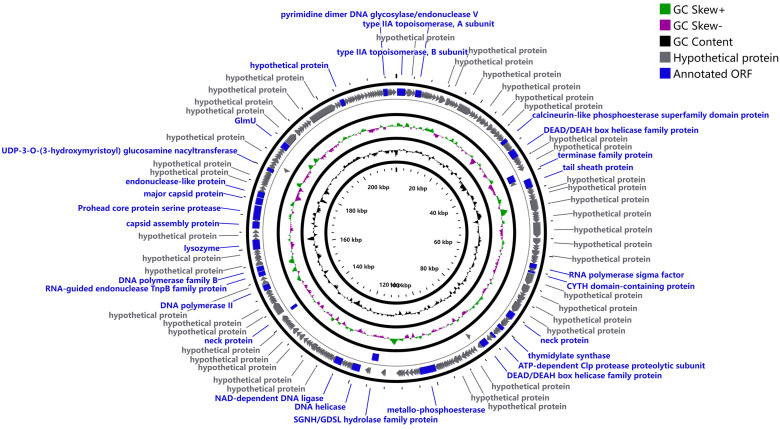
Whole-genome map of phage MA9V-2.

The genome of phage MA9V-2 has been uploaded to the NCBI database with the accession number OR513085. Sequencing results indicated that the MA9V-2 genome is a linear double-stranded DNA (dsDNA) molecule with a total length of 218,539 bp and a GC content of 36.23% (Figure 3). The genome contains 269 open reading frames (ORFs), with 263 ORFs on the plus strand and 6 ORFs on the minus strand. The longest and shortest ORF protein-coding sequences are 7104 bp and 102 bp, respectively, encoding a putative protein of 2368 amino acids and a putative protein of 34 amino acids. According to BLAST+ (v2.17.0) results, 50 of the ORF proteins showed homology to genes encoding known functional proteins, of which 19 were clustered into three modules involved in phage structure, host lysis, and DNA replication (Supplementary Table S1). The remaining 31 genes encode proteins with other biological functions. Furthermore, the MA9V-2 genome lacks integrase, indicating that it is not a temperate phage. Additionally, no tRNA genes, lysogeny genes, antibiotic resistance genes, or virulence genes were found in the MA9V-2 genome, suggesting that phage MA9V-2 may be a promising candidate for phage therapy. and holds promise as a biocontrol agent against *C. indologenes* MA9 pathogenic bacteria.

### 3.5. Genomic Synteny Analysis

**Figure 4:**
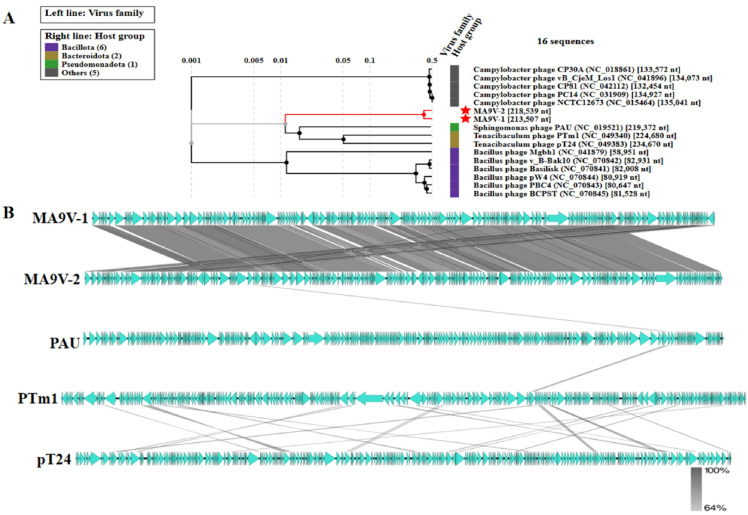
(**A**) A rectangular proteome tree constructed with the whole genome of MA9V-2 and (**B**) Multilinear genomic comparison of phage MA9V-1 with MA9V-2 and other phages. These red stars represent the target strains, and the red lines indicate the positions of the target strains on the proteome tree (**A**). The color intensity from light gray to dark gray represents the percentage level of amino acid sequence similarity (64–100%) (**B**).

Phage MA9V-2 and MA9V-1 were clustered together in the constructed rectangular protein phylogenetic tree, showing the highest gene sequence similarity. Like MA9V-1, MA9V-2 has relatively low similarity with *Sphingomonas* phage PAU, *Tenacibaculum* phage PTm1, and *Tenacibaculum* phage pT24, which are located on different branches of the same node (Figure 4A). Multiple linear alignments of MA9V-1, MA9V-2, and three other similar genomes revealed that MA9V-1 and MA9V-2 have high similarity, with most of the ORFs showing over 64% similarity, some even reaching 99%. However, the overall amino acid sequences are not identical. These two phages exhibit substantial differences from the other three, with similarity dropping to below 5% (Figure 4B), indicating that both are new phages targeting the pathogenic bacterium MA9. Taken together, based on genome similarity metrics and comparative genomic analyses, MA9V-1 and MA9V-2 can be inferred to be two new isolated phages.

### 3.6. Genomic Phylogenetic Analysis

To investigate the reason for the different infection efficiencies of the two phages MA9V-1 and MA9V-2 on the pathogenic bacterium *C. indologenes* MA9, the complete genomes of both phages were analyzed. It was found that both phages possess four highly conserved functional proteins: polymerase II, capsid assembly protein, lysozyme RrrD (lytic enzyme), and terminase family protein. Through amino acid sequence alignment, the similarities of these proteins between the two phages were found to be 98.59%, 96.67%, 96.47%, and 98.30%, respectively. These highly conserved proteins are closely related to the ability of both phages to infect *C. indologenes* MA9. Furthermore, although both phages contain genes encoding tail sheath proteins, the amino acid similarity is only 91.78%, which may be the main reason for the difference in their lytic capabilities.

**Figure 5:**
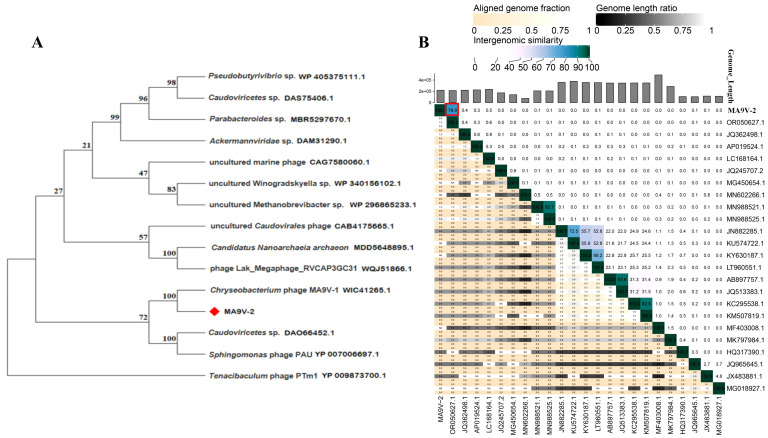
(**A**) Phylogenetic tree based on phage tail sheath protein amino acid sequences and (**B**) The VIRIDIC similarity heat map constructed by the whole genome of phage MA9V-2 and MA9V-1. The red diamond represents the target strain (**A**); The “red box” represents the similarity between genomes (**B**).

Phage tail sheath proteins are an important component of the phage tail structure. Studying these proteins helps to understand the infection mechanisms and host specificity of phages. To investigate the reason for the differences in infection between the two phages, we constructed a phylogenetic tree using the amino acid sequence of the phage tail sheath protein ORF 055 from MA9V-2 and ORF 001 from MA9V-1, along with 15 similar sequences retrieved from the NCBI database. The result, shown in Figure 5A, indicates that phages MA9V-1 and MA9V-2 cluster within the same branch with 100% bootstrap support, suggesting that the amino acid sequences of their tail sheath proteins are highly similar. However, the amino acid sequence alignment reveals a similarity of only 91.78%, which might be the primary cause of their differences in lytic ability. Additionally, the VIRIDIC similarity heatmap (Figure 5B) shows that the nucleotide sequence similarity between the two phages is 79% (<95%), indicating that they represent two different species of phages within the same genus targeting *C. indologenes* MA9. Since the two phages differ significantly from each other, they could potentially be used in combination for pathogen control research.

### 3.7. Pathogenicity Analysis of MA9 on Panax notoginseng Pot Plants

The pathogenicity of *C. indologenes* MA9 on *P. notoginseng* root segments has been previously validated in prior studies [9]. This study focuses on determining whether MA9 is pathogenic to *P. notoginseng* pot plants and the optimal pathogenic dose.

**Figure 6:**
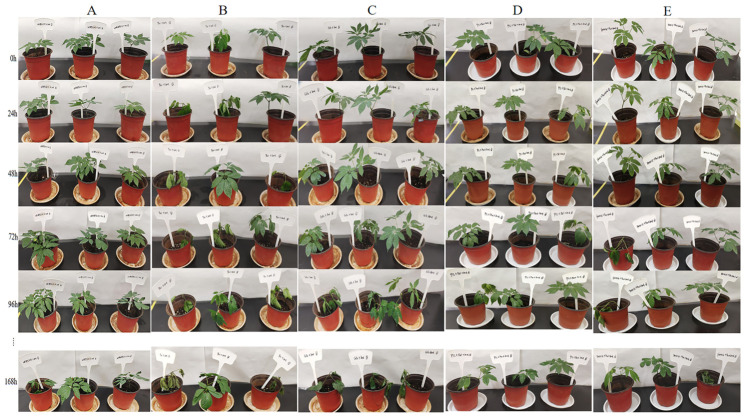
Pathogenicity assay of MA9 on live potted *P. notoginseng*. (**A**) Addition of 20 mL sterilized exponential-phase MA9 bacterial suspension as a negative control. (**B**) Spraying with 20 mL exponential-phase MA9 bacterial suspension. (**C**) Root irrigation with 20 mL exponential-phase MA9 bacterial suspension. (**D**) Spraying with 20 mL exponential-phase *Pseudomonas syringae* PSS suspension as a control. (**E**) Spraying with 20 mL exponential-phase *Bacillus cereus* BYM 41-22 suspension as a control.

After treatment with different volumes of inoculum, the results showed that a volume of 20 mL produced the most pronounced experimental effects; therefore, only the pathogenicity results obtained with the 20 mL treatment are presented in Figure 6. As shown in Figure 6A (NB control group), with increasing amounts of sterilized MA9 culture, the *P. notoginseng* plants exhibited slight wilting but generally remained healthy, indicating that the sterilized MA9 culture did not cause significant damage and was safe for the plants. In contrast, the results of the spray inoculation group (Figure 6B) and the root irrigation group (Figure 6C) demonstrated that strain MA9 was able to infect *P. notoginseng* and exhibited strong pathogenicity. Both spray and root irrigation treatments resulted in obvious disease symptoms, and according to the disease incidence grading standard, the pathogenicity of both methods reached 83.33%, indicating a high virulence. Taking all factors into consideration, it was decided to adopt the method of spraying the leaves as the means of treating the potted plants. Meanwhile, the control strain did not cause any disease symptoms in *P. notoginseng* plants (Figure 6D,E). In summary, a 20 mL volume of bacterial suspension at a concentration of 5.0 × 10^8^ CFU/mL was selected as the optimal inoculation dose for subsequent disease control experiments.

### 3.8. In Vitro Lysis Assay of Phage and Biofilm Formation Inhibition Assay

**Figure 7:**
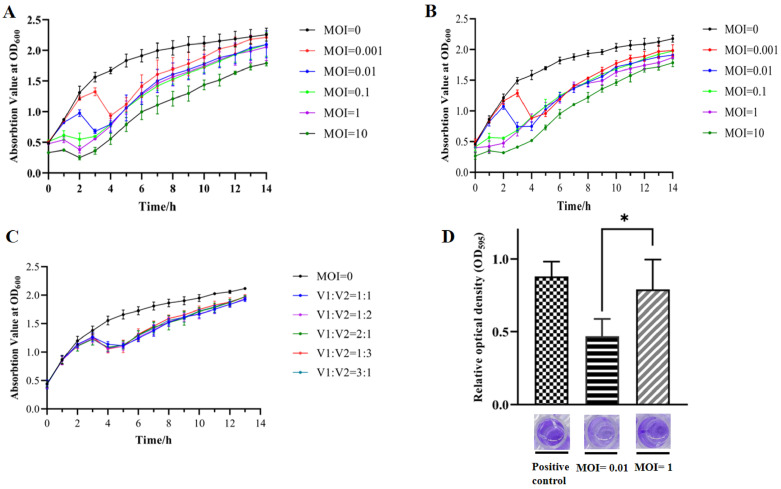
In Vitro Lysis Assay of Phage and Biofilm Formation Inhibition Assay. (**A**) In vitro lysis curve of phage MA9V-2 against host cells. (**B**) Lysis curve of the phage mixture MA9V-1 and MA9V-2 (1:1) against host cells. (**C**) Lysis curves of host cells treated with different ratios of two phages (MOI = 0.01). Data are presented as mean ± SD from three independent replicates (n = 3). (**D**) Inhibitory effect of phages on biofilm formation by pathogenic strain MA9. One-way analysis of variance (ANOVA) was used to assess statistical significance, * *p* < 0.05.

The in vitro lysis curve of phage MA9V-2 was measured. As shown in Figure 7A, the addition of phages at different MOI values inhibited the growth of *C. indologenes* MA9 to some extent, and this inhibition continued until the end of the lysis curve measurement, with the strongest inhibition at MOI = 0.01.

Furthermore, to assess the impact of combined phages on the pathogenic bacterium MA9, an in vitro lysis curve was used as a preliminary experiment. Under the condition of phage MA9V-1:MA9V-2 (1:1), the host lysis curve was compared to the lysis curve of a single phage. As shown in Figure 7B, at MOI = 0.001 and 0.01, the lysis curves showed significant differences compared to others (*p* < 0.01). In these two conditions, the lysis curve exhibited a sharp decline followed by a plateau period of approximately 1 h, before rising again at a certain rate, indicating that the combined use of two phages is more effective than the use of a single phage. Additionally, the host lysis curve at MOI = 0.01 remained consistently below the curve at MOI = 0.001, showing better lysis performance at MOI = 0.01. Thus, MOI = 0.01 was selected as the optimal condition for combining the two phages.

Building on the determination of MOI = 0.01, the mixing ratio of the two phages was further optimized. Five different ratios of MA9V-1:MA9V-2 were tested: 1:1, 1:2, 2:1, 1:3, and 3:1. The results (Figure 7C) show that, compared to the 1:1 ratio, the other ratios did not show better inhibition effects. The 1:1 ratio exhibited more noticeable inhibition, with the absorbance value consistently below the other lysis curves, demonstrating moderate inhibition. Therefore, it can be concluded that the best therapeutic effect occurs when the MOI for both phages in the combined therapy is 0.01 and the ratio of MA9V-1 to MA9V-2 is 1:1.

After crystal violet staining, the OD_595_ values of the experimental groups at MOI = 0.01 and MOI = 1 were significantly lower (*p* < 0.01) compared to the positive control group after 24 h of incubation. Furthermore, under the MOI = 0.01 condition, the combination of phages (V1:V2 = 1:1) showed stronger inhibition (0.01 < *p* < 0.05) of biofilm formation by the pathogenic bacterium *C. indologenes* MA9, with significant differences in the OD_595_ values between the two groups (*p* < 0.05). This indicates that phages have a certain inhibitory effect on the biofilm formation of *C. indologenes* MA9 (Figure 7D).

### 3.9. Composite Phage Prevention of P. notoginseng Root Rot Disease

**Figure 8:**
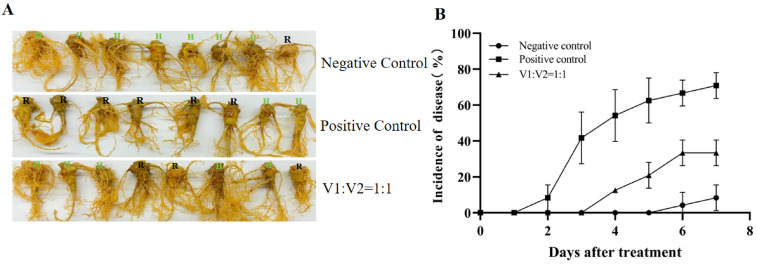
(**A**) Evaluation of combined phage treatment in preventing root disease in *P. notoginseng* of group 1:1 and (**B**) Disease incidence statistics in *P. notoginseng* under combined phage treatment. Incidence curves are shown under ratio of 1:1 (upright triangle), Positive control (square), Negative control (circle) “R” represents rotting *P. notoginseng* roots, and “H” represents healthy roots. Disease incidence was calculated as the percentage of rotten roots relative to the total number of plants in parallel experiments. Data are presented as mean ± SD from three independent replicates (n = 3).

The study of single phage prevention of *P. notoginseng* root rot disease has been previously conducted, and the results showed that single phages have a certain inhibitory effect (*p* < 0.01) on root rot, reducing the disease incidence of *P. notoginseng* roots by more than 50% [22]. The MOI for prevention was determined to be 0.01. Building on this, a prevention experiment was carried out using five different phage ratios: 1:1, 1:2, 2:1, 1:3, and 3:1 (result of 1:1 was shown in Figure 8A, other groups were shown in Supplementary Figure S1), compared to the positive control group (which received the MA9 culture in the exponential growth phase), after continuous treatment for 7 days, the disease incidence in each experimental group was very low (ID < 40%), with the lowest incidence reduced to below 50% (Figure 8B). The mixed phage solutions at different ratios delayed the onset of the disease, which further confirmed the phages’ preventive capability. Among the experimental groups, the treatment with a phage ratio of MA9V-1 and MA9V-2 at 1:1 and 1:2 showed the best results, but there was no significant difference between them. For the subsequent *P. notoginseng* pot plant biocontrol experiments and based on the results of the composite phage in vitro lysis assays, the optimal biocontrol conditions were selected as MA9V-1:MA9V-2 = 1:1 (MOI = 0.01).

### 3.10. Phage Prevention of P. notoginseng Pot Plant Disease

**Figure 9:**
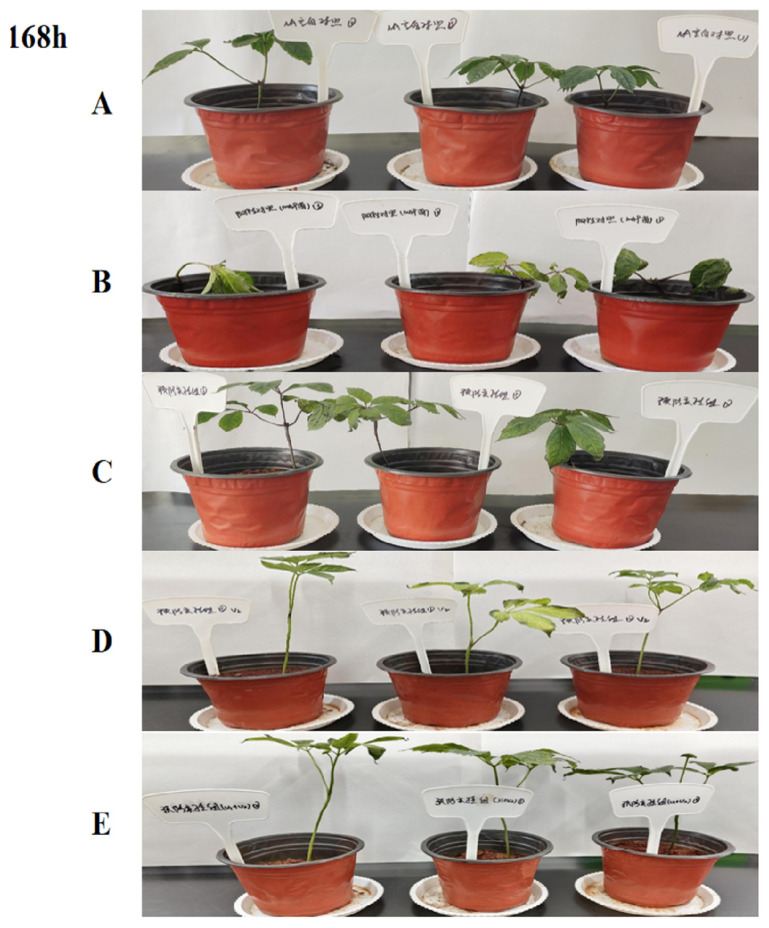
Prevention experiment results of *P. notoginseng* root rot disease treated with different methods after 168 h treatment. (**A**) Spraying with 20 mL of NB liquid medium as a blank control. (**B**) Spraying with 20 mL of MA9 bacterial suspension as a positive control. (**C**) Spraying with 20 mL of a mixture of phage MA9V-1 and host MA9 at MOI = 0.01 as a preventive treatment group. (**D**) Spraying with 20 mL of a mixture of phage MA9V-2 and host MA9 at MOI = 0.01 as a preventive treatment group. (**E**) Spraying with 20 mL of a 1:1 mixture of phages MA9V-1 and MA9V-2 (combined with host MA9 at MOI = 0.01) as a preventive treatment group.

Figure 9 only shows the growth conditions of each group after 168 h of treatment, and other results for different periods can be seen in Supplementary Figure S2. In the prevention experiment, the *P. notoginseng* plants in the experimental groups showed lighter disease symptoms compared to those in the positive control group (Figure 9C–E). In the positive control group, the disease symptoms on the plants were very obvious, with even necrosis observed (Figure 9B). In contrast, the experimental groups showed only mild symptoms, such as yellowing and drying of the leaves, indicating that the phages had a preventive effect. Among the experimental groups, the MA9V-1 prevention group showed slight lodging symptoms in the roots and stems, while the MA9V-2 prevention group showed yellowing of the leaves. However, in the combined treatment group of MA9V-1 and MA9V-2, no obvious disease symptoms were observed on the *P. notoginseng* plants. When applied to control the MA9-induced disease, the combination of both phages demonstrated better preventive effects (*p* < 0.01)than the single phages, with the incidence rate dropping by approximately 10–25%.

### 3.11. Phage Biocontrol of P. notoginseng Pot Plant Disease

**Figure 10:**
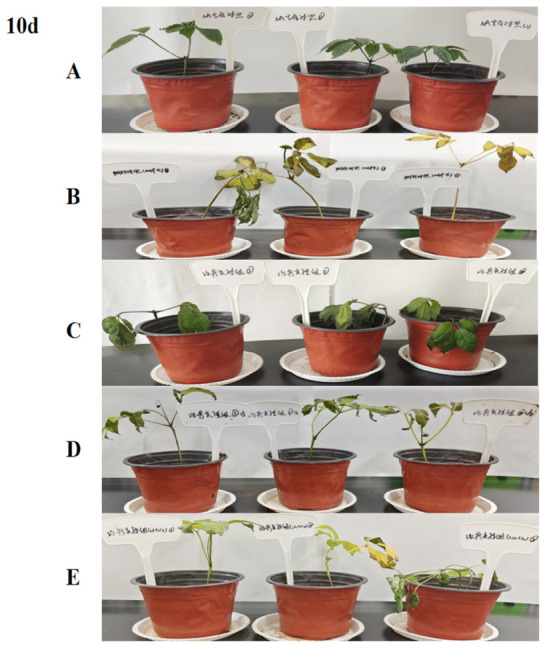
Biocontrol experiment results of *P. notoginseng* root rot disease treated with different methods after 10 d treatment. (**A**) Spray 20 mL NB liquid medium as a blank control. (**B**) *P. notoginseng* plants were first treated by spraying logarithm long-term MA9, and then sprayed with sterilized MA9 bacterial solution at the growth stage as a positive control group. (**C**) After spraying MA9 at the logarithmic growth stage to cause disease in plants, a 20 mL mixture of phage MA9V-1 and NB medium was sprayed as the treatment group. (**D**) After spraying MA9 at the logarithmic growth stage to cause disease in *P. notoginseng* plants, 20 mL of phage MA9V-2 mixed with NB medium was sprayed as the treatment group. (**E**) The plants were first infected by being sprayed with s MA9 in logarithmic growth phase, and then sprayed with 20 mL phage MA9V-1:MA9V-2 (under the condition of MOI = 0.01) at a ratio of 1:1 and mixed with NB liquid medium as the treatment group.

Similarly, Figure 10 only shows the growth conditions of each group after 10 days of treatment, and other results for different periods can be seen in Supplementary Figure S3. In the *P. notoginseng* root rot disease biocontrol experiment, the positive control group started with infected *P. notoginseng* plants, which were then treated with sterilized MA9 culture in the exponential growth phase. Compared to the disease symptoms of wilting, yellowing, and drying observed in the plants of the positive control group (Figure 10B), the experimental groups displayed different symptoms after 10 days of treatment (Figure 10C–E). In the MA9V-1 treatment group, all the plants exhibited wilting. In the MA9V-2 treatment group, the plants did not wilt but showed yellowing and drying of the leaves. After combined phage treatment, both wilting and leaf yellowing occurred, but the disease symptoms were less severe than those observed with the individual treatments. From the experiment, it was found that even when phages were applied promptly after the plants showed mild symptoms, the plants could not recover to a healthy growth state. However, the phages effectively delayed the progression of the disease. Compared to the prevention experiment, the disease control ability in the biocontrol treatment groups was not ideal. This might be related to the severity of the disease caused by the pathogenic bacterium. Once the plants are infected, it is difficult to control the disease with phages, as the plants have already lost their ability to absorb water and nutrients. Therefore, once the disease develops, it becomes uncontrollable, and phages can only slow down the progression of the disease.

### 3.12. Determination of Pathogenic Bacteria and Phage Quantities

**Figure 11:**
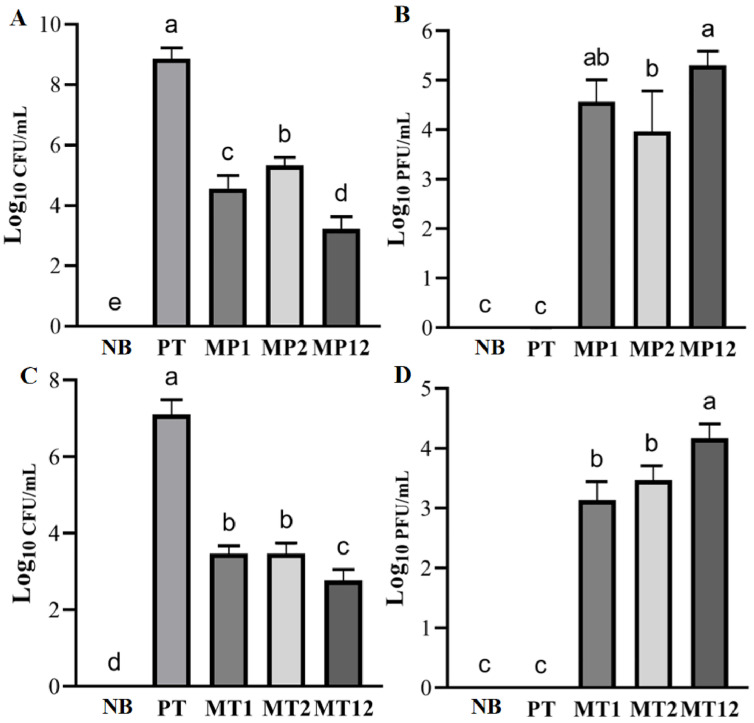
The number of pathogenic bacteria (**A**,**C**) and phage (**B**,**D**) in the leaves of *P. notoginseng* in the prevention group (**A**,**B**) and biocontrol group (**C**,**D**) was determined. Different letters above the bars indicate statistically significant differences among groups (One-way ANOVA with Tukey’s post hoc test, *p* < 0.05), while there was no significant difference for the same letters. (NB: blank control group (NB liquid medium); PT: positive control group (MA9 infection); MP1: preventive treatment with phage MA9V-1; MP2: preventive treatment with phage MA9V-2; MP12: combined preventive treatment with phages MA9V-1 and MA9V-2; MT1: treatment with phage MA9V-1; MT2: treatment with phage MA9V-2; MT12: combined treatment with phages MA9V-1 and MA9V-2).

The ability of phages to successfully infect pathogenic bacteria is crucial for their role in plant disease control. Therefore, we measured the number of phages and pathogenic bacteria in the leaves under different treatment conditions. The results from the *P. notoginseng* pot experiment showed that in the positive control group, the number of pathogenic bacteria was approximately 10^8^ CFU/mL. After different treatments, the number of pathogenic bacteria in the other experimental groups showed significant differences compared to the control group (*p* < 0.01). Among them, the preventive groups MP1, MP2, and MP12 had higher numbers of pathogenic bacteria, quantities of approximately 4.2, 5, and 3.5 (Log_10_), respectively, than the control group (*p* < 0.01). This may be due to the daily addition of phage-pathogen mixtures, whereas in the biocontrol groups, only phages were added subsequently, leading to lower pathogenic bacteria counts compared to the preventive groups. However, this did not directly correlate with the biocontrol effectiveness, indicating that the efficacy of phage treatment is not related to the number of pathogenic bacteria (Figure 11A,C).

In the phage titer measurement experiment (Figure 11B,D), the control group, which did not receive phages, showed no detectable MA9 phages. In the three preventive experimental groups, the continuous presence of the pathogenic bacteria allowed the phages to infect the host and undergo replication. However, in the biocontrol groups (The values of the phage Log_10_ are approximately 4.3, 3.8, and 5.4, respectively), the reduction in host bacteria led to a corresponding decrease in phage numbers (*p* < 0.01). Yet, since phages were added daily, their numbers remained relatively stable, showing no significant differences across the six different treatment methods (*p* > 0.05). This suggests that in the presence of phage concentration, the preventive groups had stronger disease prevention capabilities than the biocontrol group. Once the disease symptoms developed, the number of phages did not result in recovery but only slowed the progression of the disease.

### 3.13. Incidence and Biocontrol Rate Statistics

**Figure 12:**
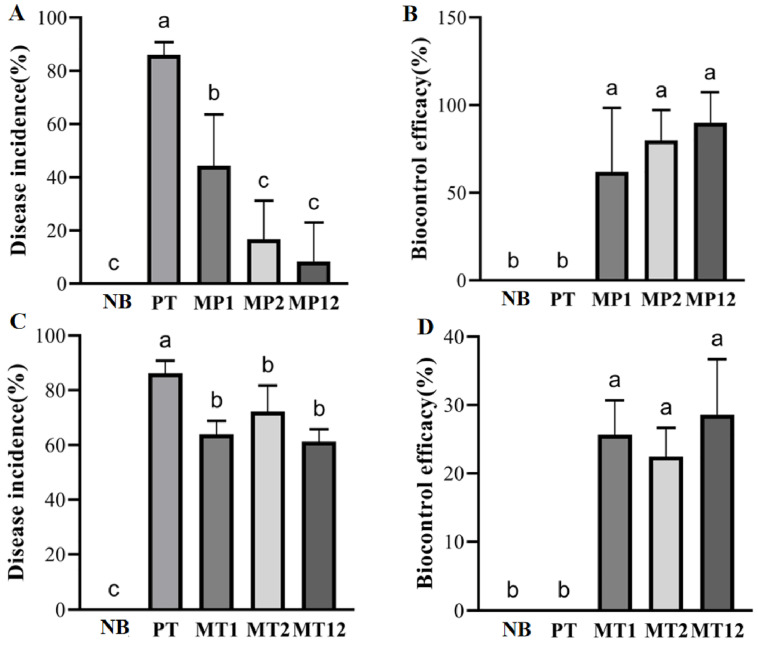
Statistics of disease incidence (**A**,**C**) and biocontrol efficacy (**B**,**D**) of *P. notoginseng* disease prevention group (**A**,**B**) and biocontrol group (**C**,**D**) (*p* < 0.05). Different letters above the bars indicate statistically significant differences among groups (One-way ANOVA with Tukey’s post hoc test, *p* < 0.05), while there was no significant difference for the same letters. (NB: blank control group (NB liquid medium); PT: positive control group (MA9 infection); MP1: preventive treatment with phage MA9V-1; MP2: preventive treatment with phage MA9V-2; MP12: combined preventive treatment with phages MA9V-1 and MA9V-2; MT1: treatment with phage MA9V-1; MT2: treatment with phage MA9V-2; MT12: combined treatment with phages MA9V-1 and MA9V-2).

The results from the *P. notoginseng* pot experiment using phage combination therapy showed that all six different phage treatments effectively controlled the occurrence of root rot disease. In the preventive groups MP1, MP2, and MP12, the disease incidence after phage treatment was 33.33%, 25%, and 25% (*p* < 0.05, one-way ANOVA with Tukey’s post hoc test), respectively (Figure 12A), significantly lower than the 83.33% in the control group, and also lower than the disease incidence in the biocontrol groups, which were 66.7%, 66.7%, and 58.3%, respectively (Figure 12C). There was no significant difference in disease incidence within the same control group, indicating that the phage concentration does not directly affect the control effectiveness. In terms of biocontrol rate, the preventive experiment showed the most significant differences (*p* < 0.01), with MP12 achieving the highest biocontrol rate of around 80% (Figure 12B). The biocontrol groups had less noticeable biocontrol rates, around 25% (Figure 12D), but still showed some control over the root rot disease compared to the control group.

## 4. Discussion

*Panax notoginseng* is a high-value medicinal crop in Yunnan Province, and root rot caused by *Chryseobacterium indologenes* MA9 poses a critical threat to its cultivation, often leading to yield losses exceeding 70% and even total crop failure. While previous studies have focused primarily on fungal pathogens, our results emphasize the significant role of bacterial pathogens in disease progression. Pathogenicity assays confirmed the high virulence of MA9, with disease incidence reaching 83.3% under optimized inoculation conditions (20 mL of 5.0 × 10^8^ CFU/mL). Leaf spraying was selected as the infection route to reflect practical field conditions and enable reproducible assessment of disease progression and severity. It is worth noting that this study included only three biological replicates (independent pots) in the pathogen infection and phage treatment experiments, which somewhat limits the statistical robustness of conclusions regarding disease incidence and biocontrol efficacy. Although the number of biological replicates per treatment was limited, the experimental results still demonstrated clear treatment differences, providing a reference for subsequent large-scale greenhouse and field experiments, as well as guidance for further studies on *P. notoginseng* root rot disease.

A new lytic phage, vB_CinP_MA9V-2, was isolated using *C. indologenes* MA9 as the host. During initial screening, plaque formation was inconsistent, often disappearing during enrichment. Optimization of culture conditions using glucose-free NA medium restored stable plaque formation, highlighting the importance of host physiological state in phage screening. MA9V-2, along with phages such as *Clostridium perfringens* IME-JL8, *Vibrio* FE11P, and *Pseudomonas aeruginosa* PPAY, was isolated from sewage, highlighting that sewage is a rich resource for phages [36,37,38]. MA9V-2 forms small plaques with a diameter of 0.4–1.4 mm on the *C. indologenes* MA9 biofilm, smaller than those formed by *Enterococcus faecalis* EFap02 and *Vibrio parahaemolyticus* GHSM17 (approximately 2 mm). Morphologically, *C. indologenes* MA9 phages are similar to *T. maritimum* phages PTm1 and PTm5, with an icosahedral “head,” contractile “tail,” and relatively short “neck”. Interestingly, although all these phages belong to the Myoviridae family, TEM imaging clearly showed flexible fibrous appendages of about 50–100 nm in length on the upper region of the heads of PTm1 and PTm5, which were absent in MA9V-2 [39]. In addition, the contractile tail undergoes a conformational change when the phage attaches to the host cell, forming a channel through which the phage genome can be injected into the host cytoplasm [40]. Phage specificity, as demonstrated by the host lysis spectrum, is one of the key factors in applying phages as alternatives to antibiotics, medical formulations and agricultural biocontrol. This characteristic ensures that phage therapy does not directly affect the density, richness, and survival of other microorganisms in the microecosystem.

Phage genome analysis is an important means to understand the phage at the genetic level. Genomic safety analysis is also crucial for phage research [41]. The whole-genome sequencing results show that the MA9V-2 genome is 218,539 bp in length, with a GC content of 36.23%, and contains 269 ORFs, encoding 50 known functional proteins. The MA9V-2 genome is shorter than that of *Pseudomonas* phage D6 (307,402 bp), which causes tomato wilt [42], and longer than that of *Xanthomonas* phage (44,044 bp) that causes black rot in cabbage [43]. Based on the detailed functional annotation of phage proteins (Supplementary Table S1), several of the proteins encoded by the phage genome are involved in DNA replication, which likely explains its ability to infect and replicate. Additionally, most of the ORFs in the phage genome are hypothetical proteins with unknown functions, possibly due to a lack of records for such phage-related functional genes in viral databases. Bioinformatic analyses, including VIPtree circular and rectangular phylogenetic tree construction, genome multiple linear alignments, and VIRIDIC similarity heatmaps, indicate that although MA9V-1 and MA9V-2 are new phage groups targeting *C. indologenes* MA9, there are differences in their infection abilities. Studies have shown that phages are natural carriers for the transfer of virulence and antibiotic resistance genes [44]. Therefore, comprehensive genome analysis of the phages in this study using VFDB software(v2024) found no virulence or antibiotic resistance genes, indicating that MA9V-1 and MA9V-2 have potential use in biocontrol without introducing risks associated with these genes.

In vitro experiments demonstrated that the optimal MOI of 0.01 allowed both phages to efficiently infect *C. indologenes* MA9, with MA9V-2 achieving 75% adsorption within 25 min and a burst size of ~100 PFU per cell. The combined application of MA9V-1 and MA9V-2 (1:1 ratio) slowed bacterial growth more effectively than single-phage treatments (Figure 7B–D) and inhibited biofilm formation to a greater extent, indicating a synergistic effect. Pot experiments further confirmed these findings: preventive application of the phage cocktail reduced disease incidence by ~10–25% compared to single-phage treatments and delayed symptom progression (Figure 8, Figure 9, Figure 10, Figure 11 and Figure 12). Compared with other phage therapy experiments, the treatment effect was better. For example, phages like RsoM1USA, isolated from tomato fields in Florida, have effectively slowed the wilting of *Ralstonia solanacearum*-infected tomato plants [14]. CFU counts showed that preventive treatments significantly reduced pathogen loads in leaves within 15 min of phage application (Figure 11A,C), demonstrating that timely host–phage interactions are critical for maximizing biocontrol efficacy. While severely infected plants could not be fully restored, phage treatment effectively delayed disease development, highlighting its potential as a preventive strategy.

It is worth noting that, for the following reasons, this paper selects the leaf spraying method as the means of treating the plants: (1) In the experiment, we found that whether the plants were treated by spraying or by root irrigation, the degree of lesion and the incidence rate of the disease of *P. notoginseng* were very close. Considering that in order to ensure that the liquid containing pathogenic bacteria is evenly covered on the sample to eliminate experimental errors. Therefore, spraying was chosen as the treatment method for the plants. And experiments have shown that the related substances of the experiment have a systemic movement within the plants [45], which may also be the cause of the correlation between leaf and root rot lesions; (2) Due to its ease of use and scalability, foliar spraying is often the preferred application method in agricultural settings. Demonstrating efficacy through foliar application enhances the practical relevance of the study’s findings. The study by Gouveia et al. showed that foliar application of potassium phosphonate can prevent root infection by root rot pathogens, providing direct experimental evidence supporting the use of foliar sprays to control soil-borne root rot diseases [46].

Several critical challenges still need to be addressed before large-scale application can be achieved. First, host defense barriers remain a major obstacle. For instance, the capsule-thickening mechanism observed in MA9, which is induced under nutrient-rich conditions, can inhibit phage adsorption and lead to unstable infection. Notably, this mechanism was reported for the first time in the present study. Second, the mechanistic understanding remains insufficient. Although the functions of the 269 ORFs in MA9V-2, including ORF192 encoding DNA polymerase, were predicted bioinformatically, more than 90% of the putative proteins have not yet been experimentally validated, and their interaction networks with the host remain unclear. In addition, the application scenarios investigated in this study were relatively limited. Only the spraying method was evaluated, while other agronomic approaches, such as root irrigation and seed coating, were not assessed for their feasibility or efficacy. Furthermore, ecological evaluations were lacking. Specifically, the long-term effects of phage release on rhizosphere microbial communities, including antagonistic microorganisms such as *Bacillus subtilis* strain Pn1, were not monitored, and no environmental safety assessment was conducted regarding the persistence of residual phage genes in soil ecosystems. Overall, through interdisciplinary collaboration among synthetic biology, microbiome science, and smart agriculture, phage therapy holds great promise as a solution for overcoming the continuous cropping obstacles in *Panax notoginseng* cultivation, thereby promoting the green and sustainable transformation of the medicinal plant industry.

## Figures and Tables

**Table 1:** Host range of phage MA9V-2.

Strains	Lysis	EOP
*Chryseobacterium indologenes* MA9(host)	*++*	1
*Chryseobacterium indologenes* 01	−	0
*Chryseobacterium indologenes* 02	*+*	0 < EOP < 0.001
*Chryseobacterium indologenes* 03	*+*	0 < EOP < 0.001
*Chryseobacterium indologenes* 04	−	0
*Chryseobacterium indologenes* 05	−	0
*Chryseobacterium indologenes* 06	*+*	0 < EOP < 0.001
*Chryseobacterium indologenes* ATCC 29897	−	0
*Bacillus cereus* MYB 41-22	−	0
*Pseudomons. Syringae* pv. *Syringae CGMCC 1.3070*	−	0
*Escherichia coli* ATCC11303	−	0

“++”: High lytic activity; “+”: Low lytic activity; “−”: No lytic activity.

## Data Availability

The datasets presented in this study can be found in online repositories. The names of the repository/repositories and accession number(s) can be found at: (https://www.ncbi.nlm.nih.gov/nuccore/OR513085.1/, accessed on 22 March 2026), OR513085.

## Supplementary Materials

The following supporting information can be downloaded at: https://www.mdpi.com/article/10.3390/microorganisms14071423/s1, Figure S1: Composite phage prevention experiment for lesions of *P. notoginseng* roots. (A) Evaluation of combined phage treatment in preventing root disease in *P. notoginseng* and (B) Disease incidence statistics in *P. notoginseng* under combined phage treatment. Incidence curves are shown under different treatment ratios, including 1:1 (upright triangle), 1:2 (inverted triangle), 2:1 (diamond), 1:3 (hollow circle), and 3:1 (hollow square). “R” represents rotting *P. notoginseng* roots, and “H” represents healthy roots. Disease incidence was calculated as the percentage of rotten roots relative to the total number of plants in parallel experiments.Data are presented as mean ± SD from three independent replicates (n = 3). Figure S2: Prevention experiment of *P. notoginseng* root rot disease by phage. (A) Spraying with 20 mL of NB liquid medium as a blank control. (B) Spraying with 20 mL of MA9 bacterial suspension as a positive control. (C) Spraying with 20 mL of a mixture of phage MA9V-1 and host MA9 at MOI = 0.01 as a preventive treatment group. (D) Spraying with 20 mL of a mixture of phage MA9V-2 and host MA9 at MOI = 0.01 as a preventive treatment group. (E) Spraying with 20 mL of a 1:1 mixture of phages MA9V-1 and MA9V-2 (combined with host MA9 at MOI = 0.01) as a preventive treatment group. Figure S3: Experiment on treatment of *P. notoginseng* root rot. (A) Spray 20 mL NB liquid medium as a blank control. (B) *P. notoginseng* plants were first pathogenic by spraying logarithm long-term MA9, and then sprayed with sterilized MA9 bacterial solution at the growth stage as a positive control group. (C) After spraying MA9 at logarithmic growth stage to cause disease in plants, 20 mL mixture of phage MA9V-1 and NB medium was sprayed as the treatment group. (D) After spraying MA9 at logarithmic growth stage to cause disease in *P. notoginseng* plants, 20 mL of phage MA9V-2 mixed with NB medium was sprayed as treatment group. (E) The plants were first caused disease by spraying s MA9 in logarithmic growth phase, and then sprayed 20 mL phage MA9V-1:MA9V-2(under the condition of MOI = 0.01) at a ratio of 1:1 and mixed with NB liquid medium as the treatment group. Table S1: Annotated ORFs in the genome of phage MA9V-2.
