# Supplementary material for: Isolation and Characterization of a New Lytic Phage MA9V-2 Against Chryseobacterium indologenes MA9 and Its Combined Application with MA9V-1 for the Control of Panax notoginseng Root Rot
Source: Microorganisms. 2026 Jun 29;14(7):1423. doi: 10.3390/microorganisms14071423 (PMC13414440; doi:10.3390/microorganisms14071423)
Supplement: Supplementary file 1 [file microorganisms-14-01423-s001.zip › microorganisms-4365519-supplementary.pdf]

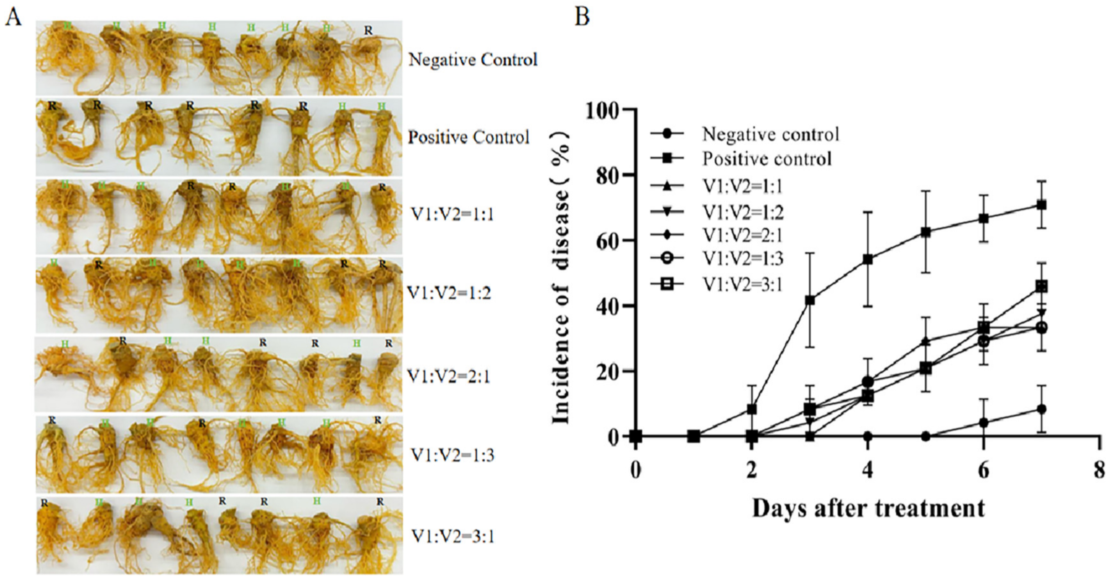

Figure S1. (A) Evaluation of combined phage treatment in preventing root disease in *P. notoginseng* and (B) Disease incidence statistics in *P. notoginseng* under combined phage treatment. Incidence curves are shown under different treatment ratios, including 1:1 (upright triangle), 1:2 (inverted triangle), 2:1 (diamond), 1:3 (hollow circle), and 3:1 (hollow square). "R" represents rotting *P. notoginseng* roots, and "H" represents healthy roots. Disease incidence was calculated as the percentage of rotten roots relative to the total number of plants in parallel experiments.Data are presented as mean ± SD from three independent replicates (n = 3)

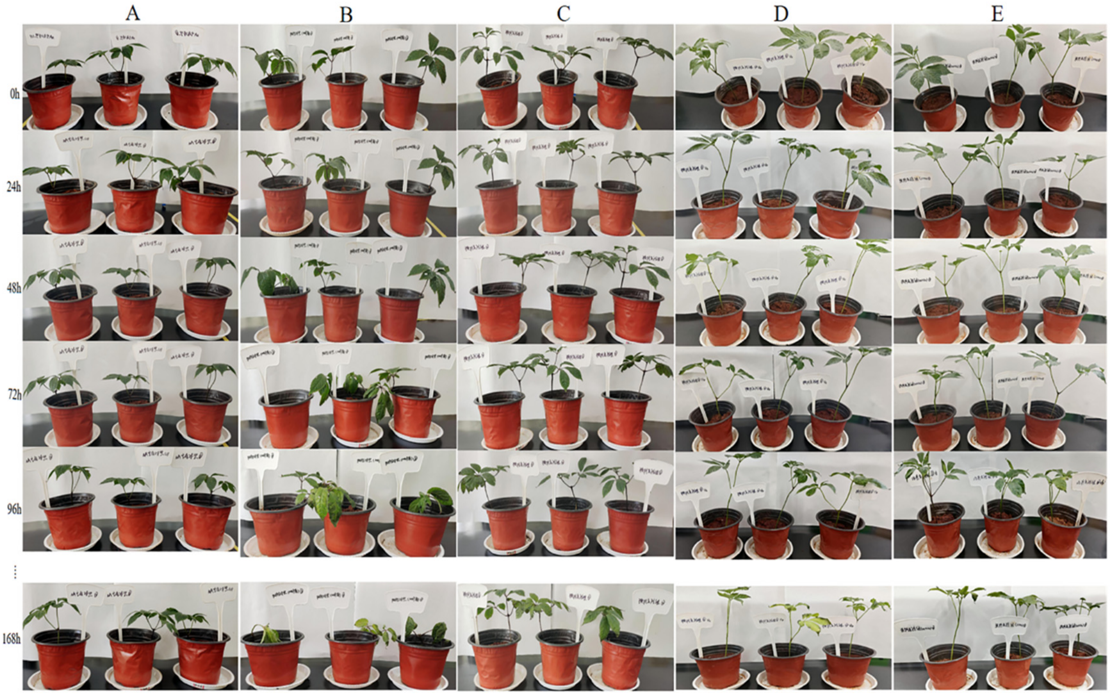

Figure S2. Prevention experiment of *P. notoginseng* root rot disease by phage. (A) Spraying with 20 mL of NB liquid medium as a blank control. (B) Spraying with 20 mL of MA9 bacterial suspension as a positive control. (C) Spraying with 20 mL of a mixture of phage MA9V-1 and host MA9 at MOI = 0.01 as a preventive treatment group. (D) Spraying with 20 mL of a mixture of phage MA9V-2 and host MA9 at MOI = 0.01 as a preventive treatment group. (E) Spraying with 20 mL of a 1:1 mixture of phages MA9V-1 and MA9V-2 (combined with host MA9 at MOI = 0.01) as a preventive treatment group.

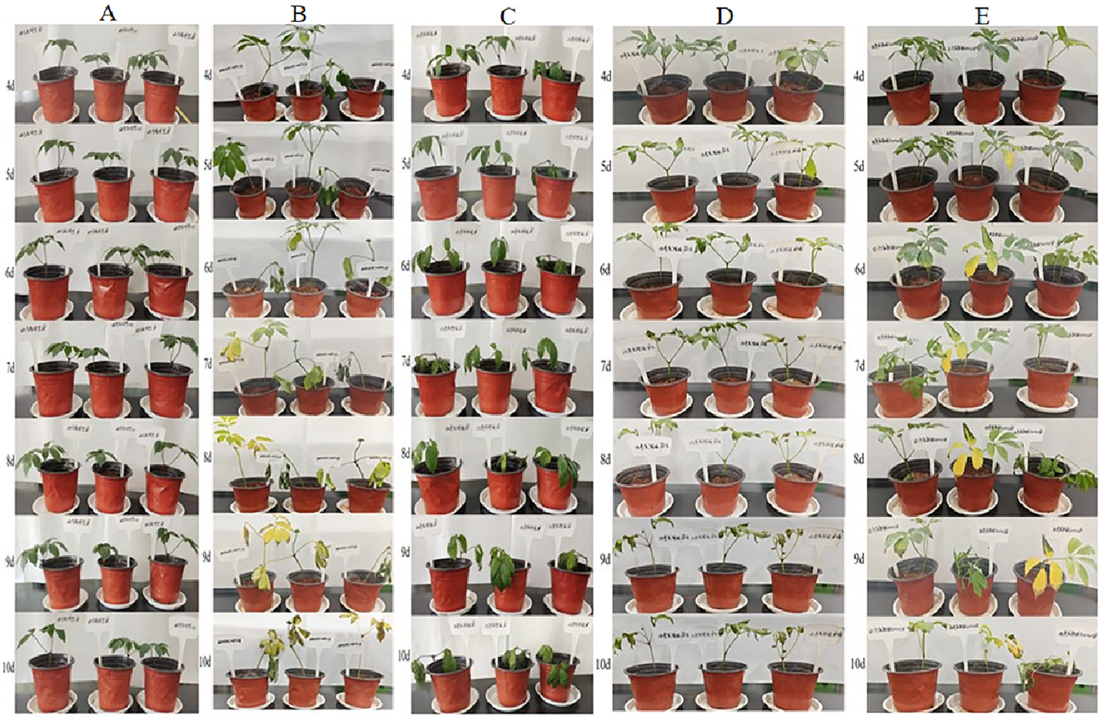

Figure S3. Experiment on treatment of *P. notoginseng* root rot. (A) Spray 20 mL NB liquid medium as a blank control. (B) P. notoginseng plants were first pathogenic by spraying logarithm long-term MA9, and then sprayed with sterilized MA9 bacterial solution at the growth stage as a positive control group. (C) After spraying MA9 at logarithmic growth stage to cause disease in plants, 20 mL mixture of phage MA9V-1 and NB medium was sprayed as the treatment group. (D) After spraying MA9 at logarithmic growth stage to cause disease in P. notoginseng plants, 20 mL of phage MA9V-2 mixed with NB medium was sprayed as treatment group. (E) The plants were first caused disease by spraying s MA9 in logarithmic growth phase, and then sprayed 20 mL phage MA9V-1:MA9V-2(under the condition of MOI=0.01) at a ratio of 1:1 and mixed with NB liquid medium as the treatment group.

Table S1. Annotated ORFs in the genome of phage MA9V-2

ORFs	Strand	Start	Stop	Length	Function	Best-match	E-values	Per. Ident	GenBank Accession. No
ORF1	+	164	2147	1983	GyrB Type IIA topoisomerase	*uncultured Caudovirales phage*	5e-119	37.85%	CAB4159554.1
ORF5	+	4652	6122	1470	GyrA Type IIA topoisomerase	*uncultured Caudovirales phage*	1e-90	38.80%	CAB4219051.1
ORF23	+	20104	20623	519	Polymer-forming cytoskeletal protein	*Streptococcus pneumoniae*	4e-43	50.62%	HEW9152019.1
ORF24	+	20632	21541	909	CapA family protein	*Bacteroidota bacterium*	6e-15	30.85%	MBU2445609.1
ORF25	+	21560	22763	1203	Cytidine and deoxycytidylate deaminase zinc-binding region	*Virus NIOZ-UU157*	7e-52	56.38%	QPI16264.1

ORF29	+	23807	24752	945	Clamp loader of DNA polymerase	*Sphingomonas phage PAU*	3e-63	36.13%	YP_007006683.1
ORF38	+	29800	30790	990	Calcineurin phosphoesterase domain, ApaH type	*uncultured Caudovirales phage*	5e-126	54.57%	CAB5218431.1
ORF45	+	32964	35100	2136	DEAD/DEAH box helicase family protein	*bacterium*	5e-82	31.35%	MDA9263013.1
ORF53	-	386721	40615	1944	Terminase-like family protein(v-2)	*Sphingomonas phage PAU*	6e-79	29.02%	YP_007006694.1
ORF55	+	41152	43339	2187	Tail sheath protein	Tenacibaculum phage PTm1	1e-37	31.95%	YP_009873700.1
ORF76	+	62344	63508	1164	Polymerase sigma factor RpoD/SigA	*gnavibacteriales bacterium*	9e-10	26.67%	MCF8305946.1
ORF103	+	85016	86774	1758	DNA helicase	Tenacibaculum phage PTm1	3e-26	24.96%	YP_009873724.1

ORF127	+	99423	103404	3981	Metallo-phosphoesterase	*Sphingomonas phage PAU.*	4e-66	24.26%	YP_007006895.1
ORF133	+	106257	107238	981	Ribonucleotide-diphosphate reductase subunit beta	*Bacteroidota bacterium*	3e-145	63.72%	MBS1572825.1
ORF139	+	110469	112191	1911	Ibonucleoside-diphosphate reductase subunit alpha	*Chryseobacterium sp.*	0	64.46%	WP_309436237.1
ORF142	+	112738	114109	1731	3-deoxy-D-manno-octulosonate 8-phosphate phosphatase	Sphingomonas phage PAU	2e-07	35.87%	WP_269392588.1
ORF147	+	118335	120375	2040	DEAD/DEAH box helicase family protein	*Candidatus Methanofastidiosia archaeon*	2e-50	27.34%	NMC59089.1
ORF154	+	123052	124972	1923	NAD-dependent DNA ligase	*Sphingomonas phage PAU*	7e-47	28.88%	YP_007006813.1
ORF164	+	129250	129703	453	GNAT family N-acetyltransferase	*uncultured Maritalea sp.*	3e-07	36.96%	WP_299346685.1

ORF170	-	133344	134334	990	3'-5' exonuclease	*Patescibacteria group bacterium*	2e-15	30.35%	MFA4941545.1
ORF174	+	138285	138606	321	Septal ring lytic transglycosylase RlpA family protein	*Moraxella lacunata*	9e-25	47.32%	WP_115004602.1
ORF192	+	149314	150802	1488	DNA polymerase II(v-2)	*bacterium*	2e-67	32.23%	MDC0008875.1
ORF196	+	154260	155538	1278	DNA polymerase domain-containing protein	*Candidatus Diapherotrites archaeon*	3e-33	28.53%	HRT03419.1
ORF203	+	159685	162154	2469	Lysozyme	*Cetobacterium sp.*	7e-34	48.95%	MEG2347381.1
ORF207	+	164908	166618	1710	Pportal protein	*Caudoviricetes* sp.	5e-73	30.78%	DAS78223.1
ORF209	+	166979	170603	3624	Prohead core protein serine protease	*Caudoviricetes* sp.	6e-23	38.27%	DAP95449.1

ORF224	+	179417	180329	912	Putative transferase, nesg, ydcK, Structural Genomics.38A	*Caudoviricetes* sp	8e-41	33.33%	DAG44881.1
ORF232	+	184054	184660	606	Dihydrofolate reductase	*Algoriphagus aestuariicola*	3e-25	43.57%	WP_206569052.1
ORF233	+	184718	185381	663	SIS domain-containing protein	*Candidatus Scatousia excrementigallinarum*	4e-27	39.29%	HIS37400.1
ORF235	+	185933	187694	1761	NTP transferase	*Caudoviricetes* sp	4e-50	27.32%	DAP95447.1
ORF249	+	206644	207253	609	PAS fold protein	*Caudoviricetes* sp.	1e-31	36.46%	DAM52910.1
ORF258	+	212304	212799	495	DUF1273 family protein	*Ignavibacteria bacterium*	2e-46	49.67%	MBK7186969.1

ORF268	-	217390	217675	285	Thioredoxin	*Flavobacterium sp.*	5e-17	38.20%	MCU0351479.1
